# Decentralized graywater treatment by a combination of sequencing batch reactor and advanced oxidation processes for reuse in Vietnam

**DOI:** 10.1002/wer.70096

**Published:** 2025-05-25

**Authors:** Stephan Beil, Amélie Chabilan, Linda Schuster, Hilmar Börnick, Minh Tan Nguyen, Stefan Stolte

**Affiliations:** ^1^ TUD Dresden University of Technology Chair of Hydrochemistry and Water Technology Dresden Germany; ^2^ Institute for R&D of Natural Products (INAPRO) ‐2 Hanoi University of Science and Technology (HUST) ‐ 1 Hanoi Vietnam

**Keywords:** advanced oxidation processes (AOP), graywater, micropollutants, sequencing batch reactor (SBR), water reuse

## Abstract

The availability of sufficient clean water has become an increasing problem even in regions with generally humid climates such as Vietnam due to rising water consumption, particularly in densely populated urban areas. The associated problems, such as an increasing scarcity of groundwater, pose major challenges for water management. Appropriate treatment and reuse of graywater, which accounts for a high proportion of total wastewater in households, can contribute significantly to solving this problem. In the present study, a combination of a biologically active sequencing batch reactor (SBR) and subsequent treatment by advanced oxidation processes (AOP) for decentralized graywater treatment is described as a promising option for water reuse in Vietnam. Treatment of synthetic graywater in a model reactor has shown that SBR is a suitable approach for efficient removal of bioavailable organic matter (BOD5 removal >95%), but that the resulting effluent does not meet the required quality criteria for reuse in terms of microbiological contamination, color and micropollutant levels. However, the subsequent AOP remedies these deficiencies. Thus, the number of coliforms could be reduced from 1320 to <1 MPN/100 mL, turbidity to <2 NTU and color by 75% to 4–6 Pt/Co‐units. With the exception of terbutryn, the graywater‐relevant micropollutants considered could be removed to such an extent by the subsequent UV/H_2_O_2_ treatment step within 60 min of treatment time that the requirements of the corresponding Environmental Quality Standard (EQS) values are met. Therefore, a combination of both methods enables efficient graywater treatment for a variety of reuse purposes.

**Practitioner Points:**

- For laboratory investigations, a synthetically produced greywater was produced on the basis of various literature references, which is representative of the Southeast Asia region under consideration.
- Aerobic biological treatment resulted in a significant improvement in water quality in terms of color and typical general wastewater parameters such as chemical oxygen demand (COD), BOD5, and ammonium.
- In contrast, the biological stage only insufficiently removed turbidity, coliforms, total P, total N, and a number of selected organic trace substances typical of greywater.
- Only subsequent treatment using a AOP process (VUV irradiation and peroxide) reduced all the parameters and studied pollutants to such an extent that the water can be reused, for example, for irrigation purposes or for groundwater recharge.

## Graphical abstract

Graywater is defined as fecal‐free, slightly contaminated wastewater from baths, showers, kitchen, or washing machines. Through a combination of biological treatment, filtration and advanced oxidation processes using UV treatment/peroxide addition, such wastewater, can be reused for various applications.

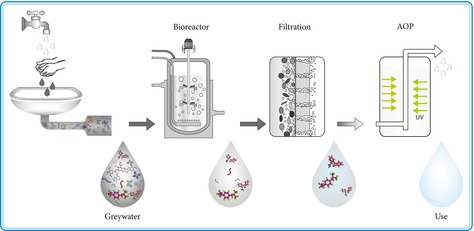

## INTRODUCTION

It is generally acknowledged that the decreasing availability of uncontaminated water, which is frequently accompanied by widespread groundwater depletion, is one of the most important global threats (Famiglietti, 2014). Even though common perception reflects predominantly on arid and semiarid regions in this context, water shortages do as well occur in regions with generally humid climate such as Vietnam due to temporal (e.g., monsoon, typhoons, and tourism) and local (e.g., conurbations vs. rural areas) discrepancies between supply and demand (Asano et al., 2007; Jimenez & Asano, 2008). Like in many other developing and emerging countries, the problem is further exacerbated by serious surface water pollution due to inadequate wastewater collecting systems and treatment facilities. The necessity to ensure a safe water supply in densely populated conurbations such as greater Hanoi area has led to excessive groundwater abstraction and soil subsidence (Le et al., 2024; Wright‐Contreras et al., 2017). Consequently, the World Bank estimated in 2008 the overall annual economic losses from poor sanitation in Vietnam to be US $780 million or 0.5% of the annual gross domestic product (GDP), whereby the extensive consumption of water resources and health issues was identified as the most important factors (Thang et al., 1995).

Water reuse offers a sustainable option for the reduction of these problems. Since reuse systems afford a direct correlation between supply and demand, they could as well help to reduce the necessity for long‐term storage capacities with the associated health issues related to, for example, mosquito nuisances (Hiwat et al., 2013) and microbial recontamination. The value of water reuse systems has been widely recognized and their implementation in larger buildings is already requested by law in many cities, for example, in Japan (Jimenez & Asano, 2008), China (Jensen & Yu, 2016), and South Korea (Noh et al., 2004). In order to enable the reuse of wastewater, efficient treatment facilities are required that afford certain water quality criteria (see Table 6), especially in order to assure the safety of people (Who, 2006). The necessary treatment obviously depends on both the intended reuse purpose and the composition of the reclaimed water.

Besides feasible common reuse applications for urban purposes (e.g., toilet flushing, air conditioning, firefighting, industrial reuse, and groundwater recharge; Asano et al., 2007), periurban irrigation for private food crops plays a very important role in Vietnam and is currently associated to health issues such as helminth infections because of a lack of proper reuse systems (Trang et al., 2007). The appropriate treatment of wastewater can be facilitated by using only partial, less‐contaminated wastewater streams of a building, which do not comprise the effluents of toilets. This so‐called graywater is much less polluted in terms of solids and microorganisms/germs. Graywater accounts for the largest share of household wastewater, ranging from 50% to 80% (Filali et al., 2022). For Vietnam, the share is even higher, at 68%–90%, possibly due to the large amount of kitchen wastewater (Büsser et al., 2007). The implementation of graywater reclamation facilities is often hampered by the nonavailability of dual plumbing systems, which are necessary to collect toilet effluents and other partial wastewater streams separately (Asano et al., 2007). But due to the prevailing use of septic tanks even in the big cities of Vietnam, the necessary separated plumbing system is already available (Schramm, 2016). At present, the collected dark graywater in Vietnam, containing partial streams from kitchen, bathroom, and washing machines, is discharged without any treatment (Ghaitidak & Yadav, 2013; Montangero et al., 2007). To enable a safe reuse for the mentioned purposes, the treatment process must address four major objectives, namely, disinfection, nutrient removal, discoloration, and, in particular for food crop irrigation, elimination of potentially harmful micropollutants.

Therefore, the present study describes the combination of a sequential batch reactor (SBR) with UV/H_2_O_2_ based advanced oxidation processes (AOP) as an efficient approach for the treatment of dark graywater. Simple filtration was performed between the two stages to remove suspended matter or microorganisms that would have a negative impact on UV treatment due to shielding and absorption of UV light. SBR is a cost‐effective method for the reduction of the organic load and major nutrients in graywater (Hernández Leal, Temmink, et al., 2010; Priyanka et al., 2022). Biochemical oxygen demand (BOD) removal is quite robust, whereas nitrification is more vulnerable and depends, for example, on the hydraulic retention time (Lamine et al., 2007). Since Vietnamese surface water and groundwater is currently heavily polluted with ammonium (locally up to 80 mg/L for groundwater in dry season [Nguyen et al., 2014]), its removal is a central aim. Graywater usually contains a broad variety of micropollutants, for example, skin applied pharmaceuticals and personal care products as well as anticorrosives or pesticides (washing of vegetables), which cannot be degraded sufficiently by SBR. AOP affords in this respect an interesting supplementary technology (Hernández‐Leal et al., 2011). For example, application of UV/H_2_O_2_ should furthermore safeguard sufficient disinfection for the intended reuse purposes. Within the scope of the presented systematic study, the removability of selected graywater‐typical micropollutants shall be demonstrated by means of a synthetically produced graywater by combining biological treatment and AOP.

## MATERIALS AND METHODS

### Materials

All chemicals used were of analytical grade. Water, acetonitrile (both Chemsolute, Th. Geyer GmbH & Co. KG) and acetic acid (LiChropur, Merck KGaA) used for LC–MS/MS measurements and the corresponding sample preparation were of LC/MS grade. A detailed list of all chemicals and standard substances used can be found in (Table S1).

### Preparation of synthetic graywater

The mass concentration of each component of the prepared graywater is specified in Table 1. The composition is intended to reflect a typical graywater composition in Vietnam and resulted from a comprehensive literature review as summarized in Table 2. For a time‐efficient preparation and to avoid interferences and degradation of the components during storage, seven independent stock solutions (2 L each) were prepared according to Table 1 using previously autoclaved tap water and stored at 4°C.

**TABLE 1:** Composition of used synthetic graywater and storable stock solutions.

Compound	β_GW_ [mg/L]	β_stock_ [g/L]	No.^†^
KNO_3_	1.6	0.18	1
MgSO_4_	100	11	2
Na_2_SO_4_	100	11	2
CaCl_2_ × 2 H_2_O	73.5	8.1	3
NaCl	120	13.2	3
NH_4_Cl	145	9.8	3
Na_2_HPO_4_	6.9	0.76	4
KH_2_PO_4_	6.6	0.73	4
Polyphosphate	23.6	2.6	4
NaHCO_3_	524.5	7.7	5
Glycerol	100	11	6
Urea	16	1.8	7
Cellulose	200	‐	*
Humic acid	50	‐	*
Kaolin	100	‐	*
SDS	50	‐	*
WWTP effluent	50 mL/L	‐	*

*Note*: β_GW_ final mass concentration in graywater, β_stock_ mass concentration in separate stock solutions, ^†^ indicates No. of separate stock solution to avoid interferences during storage, *added separately.

Abbreviation: WWTP, wastewater treatment plant.

**TABLE 2:** Characteristics of synthetic graywater as used within this study compared to reported values for Asia and Europe.

Parameter	Synthetic graywater, with micropollutants	Real graywater Asia	Real graywater Europe
pH	8.4	6.5–8.1 ^(1)^	6–9.2 ^(2,3)^
Conductivity [μS/cm]	1210	48,900–55,000 ^(4)^	7520 ^(5)^
COD [mg/L]	882	55–630 ^(1)^	49–4210 ^(2)^
TOC [mg/L]	205	‐	114–254.5 ^(5)^
DOC [mg/L]	187	‐	7.61–276 ^(2)^
BOD5 [mg/L]	210	23–370 ^(1)^	14–1700 ^(2)^
TN [mg/L]	52	24.2–57.7 ^(4,6)^	1.7–47.8 ^(5,7)^
NH_4_^+^‐N [mg/L]	29	10.3–18.7 ^(4,6)^	0.2–16.4 ^(5,7)^
NO_3_^−^‐N [mg/L]	6.6	0.5–0.67 ^(1,4)^	<0.2–0.6 ^(7)^
TP [mg/L]	6.9	4.9 ^(6)^	2.7–10.8 ^(8)^
PO_4_^3−^‐P [mg/L]	1.6	1.5–3.4 ^(4)^	<0.05–2.3 ^(5,7)^
Turbidity [NTU]	158	20.6–43 ^(1,4)^	‐
Pt/Co units	126	‐	‐
Total coliforms [MPN/100 mL]	>2400	‐	‐
*E. Coli* [MPN/100 mL]	2	‐	‐

Abbreviations: COD, chemical oxygen demand; DOC, dissolved organic carbon; Toc, total organic carbon; TP, total phosphorus.

*Source*: ^(1)^ (Ghaitidak & Yadav, 2013); ^(2)^ (Deshayes et al., 2015); ^(3)^ (Palmquist & Hanæus, 2005); ^(4)^ (Boyjoo et al., 2013); ^(5)^ (Hernández Leal, Temmink, et al., 2010); ^(6)^ (Paris & Schlapp, 2010); ^(7)^ (Noutsopoulos et al., 2018); ^(8)^ (Sievers & Londong, 2018)

For the preparation of 11‐L synthetic graywater, 100 mL of each stock solution was mixed in a 5‐L beaker containing tap water (electric conductivity: 270 μS/cm, dissolved organic carbon (DOC): 1.8–2.3 mg/L) under stirring. To this mixture, 2.2‐g cellulose, 0.55‐g humic acid, 1.1‐g kaolin, and 0.55‐g SDS were added. After filling to 5 L with tap water, the brown mixture was stirred until the humic acid had been dissolved (approximately 10 min). The graywater was transferred into a 20‐L canister; 550‐mL effluent of a local municipal wastewater treatment plant (WWTP) was added, and the whole mixture finally filled to 11 L.

To study the removal of a selection of 11 micropollutants from graywater by SBR, 10 mL of a 44‐mg/L micropollutant stock solution in 50 vol.‐% aqueous methanol were added to the graywater (final concentration of each micropollutant in the synthetic graywater: 40 μg/L). The application of methanol was necessary due to the limited water solubility of several micropollutants at the concentrations necessary to prepare a stock solution. Samples for micropollutant and further physical, chemical, and microbiological analysis were taken from the final mixture.

### Operation of the sequencing batch reactor (SBR)

The SBR with a total capacity of 50 L was operated at a total volume of 22 L. Fresh graywater was fed to the reactor daily from Monday to Friday (24‐h operation intervals including settling time of 30 min and approximately another 30 min for daily resupply of fresh graywater). To maintain a constant sludge age of 30 days, 1 L of the suspended SBR content was removed on each operation day for the determination of the settleable solids/sludge volume. After 30 min of sedimentation, further 9 L of the supernatant was removed and the reactor volume readjusted to the total volume of 22 L by addition of fresh synthetic graywater. Hence, in total, 10 L of the total volume of 22 L were replaced by 10 L of fresh synthetic graywater every 24 h. This results in a mean (theoretical) residence time of 52.8 h for the SBR, according to

$$t_{\text{residence}}=V_{\text{total}}/V_{\text{exchanged}}\times 24\;h$$

with a volume exchange rate f_A_ = ΔV/V_R_ of 0.45.

The SBR was stirred continuously, and air was supplied to the reactor using a diffuser to maintain the O_2_‐concentration at 8–9 mg/L. After an initial 14‐day setup and inoculation of the SBR, the reactor was operated as described for two additional months to stabilize the degradation rates, and operating parameters (in particular the sludge volume) before micropollutants were added to the regularly supplied graywater. For a figure and additional information on the SBR, see Figure S1.

In order to study the micropollutant removal, samples were taken from the well‐mixed reactor content daily just after graywater addition, 2 h later, and from the effluent of the SBR just before the graywater exchange of the next day. Furthermore, sludge samples were taken after sedimentation.

Activated sludge for SBR inoculation and WWTP effluent for graywater preparation were obtained from a local municipal WWTP.

### AOP using UV/H_2_O_2_

AOP degradation experiments were performed in a laboratory‐scale reactor UXPM‐LAB‐400 (UV application and electrodeless UV lamps GmbH & Co. KG), comprising a stainless‐steel tank (volume: 40 L), a pump (16/19 L/min, Iwaki Co., LTD) and a stainless‐steel UV‐reactor with a quartz tube housing the medium pressure UV lamp (400 W, uv‐technik Speziallampen GmbH). A water cooling system connected to the double‐walled tank prevents overheating. A figure of the setup as well as further technical information on the UV system used can be found in Supporting information, p. 4 and Figure S2.

The effluent of the SBR reactor was filtrated (ash‐free filter papers for quantitative and gravimetric analyses, Grade 388, 12–15 μm, Sartorius) to remove the majority of particles. Subsequently, 8 L of the filtrated effluent was transferred to an AOP reactor; 8 mL H_2_O_2_ (35%) was added and irradiated at 400 W for 1 h. Samples were taken at time intervals of 2, 4, 6, 8, 10, 15, 20, 25, 30, 35, 40, 45, 50, and 60 min for analysis.

### Measurement of pH, electric conductivity, dissolved oxygen (DO)

pH values, electric conductivity and DO were determined via online‐probes immersed into the SBR using HQ40d multi‐device (Hach Lange). Values were recorded in 15‐min intervals.

### Characterization of physical parameters (filtrate solids residue, settleable solids, turbidity, and color)

For determination of filtrate solids residue, 100 mL of the reactor volume were filtered through washed and previously weighted ash‐free filter papers (Grade 388, 10–15 μm, 110 mm, Sartorius) and dried at 105°C overnight. The filtrate solids residue content was calculated based on differential weighing (according to DIN 38409‐1:1987‐01, 1987).

Settleable solids/sludge volume was determined by phase separation of 1 L of the well‐mixed SBR content within 30 min using an Imhoff sedimentation funnel (according to DIN 38414‐10:1981‐09, 1981).

Turbidity measurements were performed using a TN‐100/T‐100 instrument (Eutech Instruments, Thermo Scientific). Each day, quality standards for 20, 100, and 800 NTU were checked prior to sample measurement and the measuring vessel was pre‐flushed with unfiltered sample.

For determination of the spectral absorption coefficients at 254 nm (SAC_254_) and at 436 nm (SAC_436_), samples were filtrated (0.45 μm, cellulose acetate, Sartorius) and measured in 10‐mm quartz cells, pre‐flushed with filtered sample, using UV/VIS spectrophotometer Cary (Varian) (according to DIN 38404‐3:2005‐07, 2005 and DIN EN ISO 7887:2012‐04, 2012). Furthermore, color values referring to the in Vietnam frequently used Pt/Co scale were determined by correlation to absorption values at 340 nm. In order to obtain such a correlation, Pt/Co scale calibration standards representing 20, 40, 60, 80, 100, 200, 300, 400, and 500 units were prepared according to DIN EN ISO 6271:2016‐05 (2016). The calibration standards were measured at 340 nm, and the following linear correlation was obtained:

(1) $$\mathrm{Abs}\left(340\;\mathrm{nm}\right)=0.291\frac{\mathrm{Pt}}{\mathrm{Co}}+0.258,R^{2}=1.000$$

### Determination of nutrient‐related parameters (TOC, DOC, COD, BOD5, TN, N‐species, TP, PO_4_^3−^)

For total organic carbon (TOC) and DOC measurements, a TOC‐V Analyzer (Shimadzu) was applied. For TOC analysis, five droplets of 2 M HCl were added to the unfiltered samples (40 mL) prior to measurement to ensure a pH lower than 4.0. In the case of DOC measurements, samples were filtrated through syringe filters (1.0/0.45 μm, glass fiber/PET, Macherey‐Nagel, Düren, Germany) before HCl addition (according to DIN EN 1484:2019‐04, 2019).

Chemical oxygen demand (COD) was determined with COD cell test (WTW) (according to DIN ISO 15705:2003‐01, 2003). The associated chemical pulping step was performed at 148°C.

BOD within 5 days (BOD5) was determined by respirometric method using the OxiTop system (WTW) according to DIN EN 1899‐1:1998‐05 (1998). Allylthiourea was added as nitrification inhibitor. Unfiltered samples were shaken for 20 min at 175 rpm prior to measurement in order to ensure oxygen saturation. Dilution water was prepared according to DIN EN 1899‐1:1998‐05 (1998). Three solid NaOH platelets were placed into the OxiTop device before bottle closing and the samples incubated for 5 days at 20°C under constant stirring.

Total nitrogen (TN) was analyzed using a TOC‐V Analyzer equipped with a TNM‐1 unit (Shimadzu) according to DIN EN 12260:2003‐12 (2003). Unfiltered samples were diluted by Factor 2 using ultrapure water and five droplets of 2 M HCl were added.

For the determination of N‐species, samples were filtrated immediately after collection (0.45 μm, cellulose acetate, Sartorius) and stored at 5°C until measurement. Ammonium nitrogen (NH_4_^+^‐N) was analyzed according to DIN 38406‐5:1983‐10 (1983) and nitrate nitrogen (NO_3_^−^‐N) according to DIN 38405‐9:2011‐09 (2011) by photometric analysis.

Total phosphorus (TP) and orthophosphate phosphorus (PO_4_^3−^‐P) were determined according to DIN EN ISO 6878:2004‐09 (2004) by reaction with ammonium molybdate and subsequent photometric analysis.

### Microbiological parameters

Total coliforms and *E. coli* were analyzed using the test kit Colilert‐18 (IDEXX). SBR effluent was filtrated through filter paper before analysis in order to remove sludge particles.

### Trace analysis of micropollutants in aqueous samples and sludge by LC–MS/MS

Five milliliters of the aqueous sample were mixed with 500 μL of potassium phosphate buffer solution (pH 7.0, 10 mM), 100 μL of aqueous EDTA solution (25 g/L) for the desorption of particle‐bound micropollutants (Rossmann et al., 2014), 50 μL of internal standards solution (100 μg/L in methanol/water, 50/50 [v/v]) and 350 μL of water. Subsequently, 4 mL of the mixture was drawn into a syringe, 1.5 mL of the sample was directly filtrated into a first HPLC vial using syringe filters of regenerated cellulose (0.2 μm, Macherey‐Nagel, Düren, Germany), and another 1.5 mL was filtrated through the same filter in a second HPLC vial. Afterwards, the filter was removed and eluted with 1‐mL acetonitrile into an evaporation vessel. The acetonitrile was removed in nitrogen stream at 40°C to 0.1 mL and the sample reconstituted in a total volume of 1 mL with water. The resulting aqueous sample was transferred into a third HPLC vial. All three sample aliquots were used for LC/MS measurements.

Sludge samples were extracted based on the procedure of Ogunyoku and Young (2014); 50 mL of the sedimented sludge of the Imhoff funnel (30‐min sedimentation period) was collected and centrifuged for 10 min at 5000 rpm (Hermle Z 383 K). Subsequently, the supernatant was discarded and the sludge resuspended in 10 mL of water (LC/MS grade). This washing step was repeated twice. The remaining sludge was dried for 24 h at 70°C; 50 mg of the dried sample were spiked with 50‐μL internal standard solution (as specified above) and extracted with 30‐mL MeOH/acetone (50/50, v/v) at 55°C for 24 h at 220‐rpm shaking and reflux (Büchi Syncore); 20 mL of the supernatant was transferred to an evaporation vessel and the solvent removed in vacuum to a remaining volume of 2 mL, which was further reduced in nitrogen stream at 40°C to 0.1 mL and diluted with water (LC/MS grade) to a final volume of 4 mL. After thorough mixing, the aqueous sample was drawn into a syringe and treated as explained above for aqueous samples.

UHPLC separation was performed using a Shimadzu system, comprising two Nexera X2 LC‐30 AD high pressure pumps, a Nexera X2 SIL‐30 AC autosampler and a CTO‐20 AC column oven. The UHPLC was used in combination with a QTRAP 6500 + MSD from Sciex for conducting the required LC–MS/MS experiments. A Kinetex EVO C18 column (100 × 2.1 mm I.D., 1.6 μm) was applied at a constant flow of 0.6 mL/min and at 40°C. For chromatographic separation, a linear gradient of eluent A (water with 0.02% acetic acid) and eluent B (acetonitrile with 0.02% acetic acid) was generated. After an initial phase of 1 min at 5% eluent B, the share of eluent B was increased linearly to 30% within 10 s, further increased to 98% within 4.1 min and kept at that level for 2.2 min before returning to the starting conditions. Detection of micropollutants was performed in ESI(−) or ESI(+) mode according to optimized ionization conditions for each analyte using the following source conditions: IS ‐4500 V (ESI(−)) or IS +5500 V (ESI(+)), Temperature 300°C, Curtain Gas 40 psi, Collision Gas medium, Gas 1, and Gas 2 at 50 psi. Time‐dependent changes were monitored by MRM measurements (see Table S1).

### Property predictions based on COSMO‐RS theory

Octanol/water partition coefficients (log K_OW_), dissociation corrected partition coefficients (Log D) and water solubilities were predicted using COSMO*therm* software (COSMOtherme, 2018) based on COSMO‐RS theory (“COSMOtherm, Version C30, Release 18”; Eckert & Klamt, 2002; Klamt, Eckert; Hornig, et al., 2002). The applied algorithm for the calculation of water solubilities involves a QSPR estimate of ΔG_fusion_ (Klamt & Eckert, 2000). DFT/COSMO calculations of the micropollutants were carried out using the software TURBOMOLE (TURBOMOLE, 2018) with BP density functional theory applying triple‐zeta valence polarization (TZVP) basis sets (“TURBOMOLE V7.3 2018”). Log K_OC_ values were predicted using COSMOquick based on a QSPR model (Loschen et al., 2016; Loschen & Klamt, 2012).

## RESULTS AND DISCUSSION

### Characteristics of synthetic graywater

The composition of graywater is rather variable according to the collected wastewater streams (e.g., whether containing kitchen effluents and washing machines or only shower and hand‐washing basins) and regional differences. Therefore, in the context of the present investigations, synthetic graywater has to represent dark graywater, which represents the predominant graywater type in Vietnam. The applied composition is based on the reports of Nghiem et al., 2006, Schäfer et al., 2006, Hourlier et al., 2010, and Abed & Scholz, 2016. Furthermore, pure chemicals were used to generate the synthetic graywater instead of typical household products in order to ensure a reproducible preparation and to avoid significant variations due to changing batch compositions of commercial household products.

Characteristics of the obtained synthetic graywater are given in Table 2 and compared to parameter ranges of real graywater reported for Europe and Asia. The corresponding parameters were adjusted to resemble more closely the values reported for Asian graywater due to the intended application in Vietnam. Moreover, the content of nitrogen and phosphorus species was chosen closer to the upper limit of the reported values for two reasons. On the one hand, the few available reports on Vietnamese graywater indicate rather high values of those parameters due to specific regional circumstances (Paris & Schlapp, 2010). On the other hand, the intended biological treatment by SBR requires a certain nutrient content for efficient performance (ATV‐DVWK, 1997; Neitzel & Iske, 1998).

The micropollutant composition in graywater differs significantly from municipal wastewater. While orally administered drugs and their human metabolites are irrelevant due to the missing toilet stream, other pharmaceuticals and substances, which are applied to the skin, for example, certain nonsteroidal anti‐inflammatory drugs (NSAID), UV filters, and insect repellents, are of major concern. Due to the impact of wastewater streams originating from shower and hand‐washing basins, personal care products and additives thereof (e.g., parabens, preservatives, and disinfectants) are rather important. In the case of dark graywater, anticorrosives from dish washers or washing machines and partially pesticides from washed vegetables and fruits or contaminated clothes have to be considered as well. Table 3 gives a summary of the micropollutants, which were selected for preparing the synthetic graywater, and compares them to reported concentration ranges, which have been determined in real graywater or, if missing, for wastewater or surface water. Most of the micropollutants were detected in concentration ranges of several μg/L. For the present study, 40 μg/L were selected for each micropollutant to mimic a high but still realistic concentration range.

**TABLE 3:** Overview of the studied micropollutants.

Micropollutant	Group	β_GW,max_^(a)^ [μg/L]	Log K_OC_^(b)^	Log K_OW_/log D^(c)^ (pH 8.4)	Water solubility^(c)^ [mg/L] (pH 8.4)
Methylparaben	Preservative	41 ^(1)^	1.54	1.58	3200
Benzylparaben	Preservative	41 ^(1)^	2.71	3.08	44
Triclosan	Preservative	16 ^(2)^	4.35	4.79	1.3
Triclocarban	Preservative	11.4*^,(3)^	4.35	4.28	5.3
Octinoxate	UV blocker	19 ^(4)^	4.03	6.01	0.5
1H‐benzotriazole	Anticorrosive	16 ^(5)^	1.17	−0.33	14,000
Bisphenol A	Plasticizer	1.2 ^(6)^	2.66	3.16	160
Diclofenac	NSAID	4*^,(7)^	3.69	0.70	5.5
DEET	Repellent	3*^,(8)^	2.12	2.46	1300
Chlorpyrifos	Pesticide	10.8^#,(9)^	4.15	5.85	0.091
Terbutryn	Pesticide	5.6^#,(10)^	3.40	4.15	5.9

*Source*: ^(a)^maximum reported concentration in graywater; ^(b)^calculated using COSMOquick (Christoph Loschen & Klamt, 2012); ^(c)^calculated using COSMOtherm which is based on COSMO‐RS theory(“COSMOtherm, Version C30, Release 18”; Eckert & Klamt, 2002; Klamt, Eckert, & Diedenhofen, 2002; Klamt, Eckert, Hornig, et al., 2002, Klamt & Eckert, 2000); *reported value in municipal wastewater; ^#^reported value in surface water; ^(1)^ (Eriksson et al., 2009); ^(2)^ (Almqvist & Hanæus, 2006); ^(3)^ (Heidler et al., 2006); ^(4)^ (Balmer et al., 2005); ^(5)^ (Turner, 2017); ^(6)^ (Hernández Leal, Vieno, et al., 2010); ^(7)^ (Langenhoff et al., 2013); ^(8)^ (Geiling, 2015); ^(9)^ (Marino & Ronco, 2005); ^(10)^ (Quednow & Püttmann, 2007)

Furthermore, a calculated log K_OC_ value is specified for each compound in Table 3. This partition coefficient allows an estimation of the adsorption behavior of those substances to activated sludge during biological wastewater or graywater treatment. According to the EPA Sustainable Futures—P2 Framework Manual (US EPA, 2012) compounds with log K_OC_ < 2.4 show only weak or even no sorption to sludge and will consequently remain predominantly in the aqueous phase during biological treatment, while compounds with log K_OC_ > 3.5 will be rapidly adsorbed to the sludge. In this regard, triclosan, triclocarban, octinoxate, and chlorpyrifos with log K_OC_ values above 4 are prone to pronounced removal by sorption to the activated sludge, while compounds like methyl paraben, 1H‐benzotriazol, and DEET will remain in the water body unless sufficiently degraded. The high log K_OC_ value for diclofenac (pK_a_ 4.77) refers to the uncharged molecule, whereas at pH 8.4 (SBR, see Table 2), diclofenac is negatively charged, which increases its hydrophilicity and reduces the adsorption potential to sludge. Accordingly, the dissociation at pH 8.4 has been taken into account when predicting log D and water solubility.

### Nutrient removal/discoloration by SBR treatment of synthetic graywater

**FIGURE 1:**
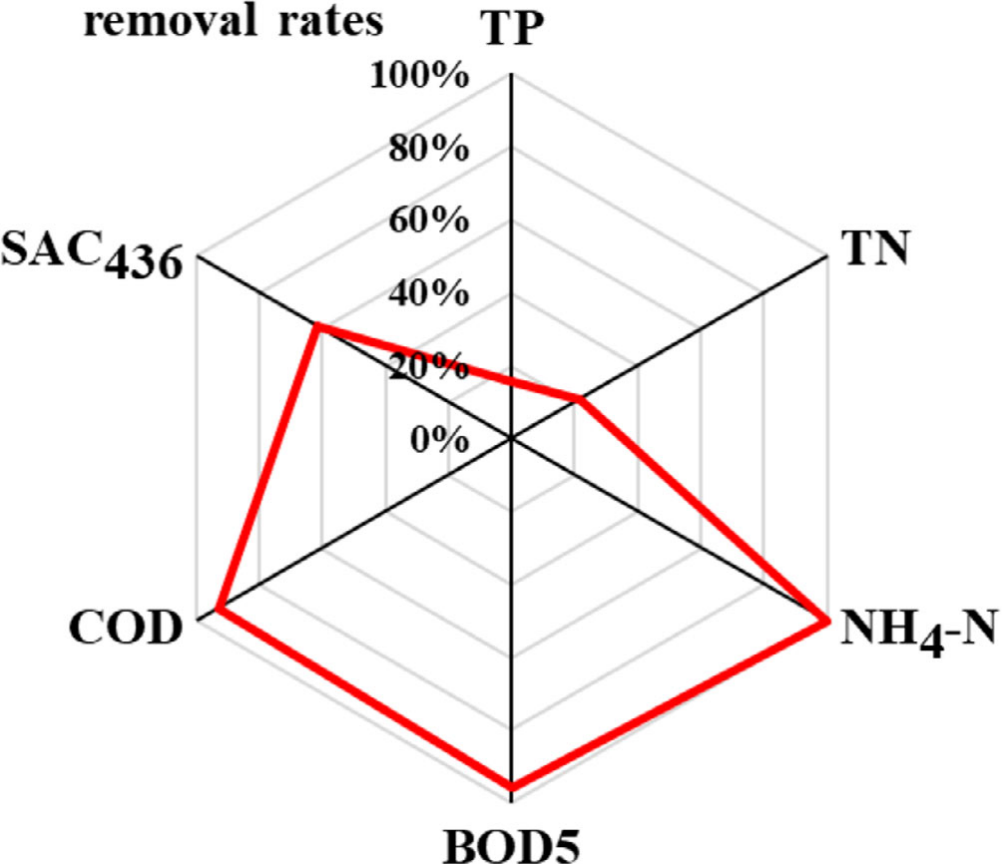
Removal rates of main nutrients and color during SBR treatment of synthetic graywater without micropollutants.

Before and after the micropollutant dosing experiment, the dry sludge content as well as settleable solids/sludge volume remained relatively constant at 1.9 +/−0.3 g/L (*n* = 4), which corroborates proper and stabile operation of the SBR. During the micropollutant dosing stage, the dry sludge content decreased significantly (1.16 g/L), whereas the sludge volume index increased (51 mL/g compared to 30.2 +/−7.3 mL/g; *n* = 4). This might indicate a reaction to the presence of the micropollutants and/or the methanol content in the stock solution solvent. However, the system rapidly recovered subsequently. The removal rates for COD and BOD5 (93% to 96%, see Figure 1) within a mean theoretical residence time of 52.8 h indicate rather efficient reduction of oxidatively degradable organic compounds. The removal of ammonium is almost complete, which implies efficient nitrification by the microorganisms of the SBR. However, TN and TP removal are significantly lower with only 21% and 16%, respectively. Thus, most probably, the denitrification step from NO_3_^−^ to N_2_ and the phosphorus removal in general are much less efficient.

The color, represented by the spectral absorption coefficient at 436 nm (SAC_436_), is reduced by more than 60% during the SBR treatment, which can be recognized by naked eyes as the dark brown color of the synthetic graywater according to its humic acid content gets much lighter during treatment. Even though, a brownish color is not necessarily associated to health issues or other detrimental properties for reuse, the perception of colored water as dirty is a major obstacle for reuse acceptance and therefore color removal an important treatment aim.

The effect of supplementing the feed graywater of the SBR with a micropollutant mixture in aqueous methanol leads to a temporary decline of the removal rates of several nutrient associated parameters (particularly COD, BOD, and ammonium removal). This effect is attributed mainly to the present methanol, which can hardly be avoided due to solubility limitations of several micropollutants in a higher concentrated stock solutions. However, in analogy to the sludge properties (see above), the removal efficiency increases again and reaches the initial level after the addition of micropollutants/methanol is stopped.

### Micropollutant elimination

**FIGURE 2:**
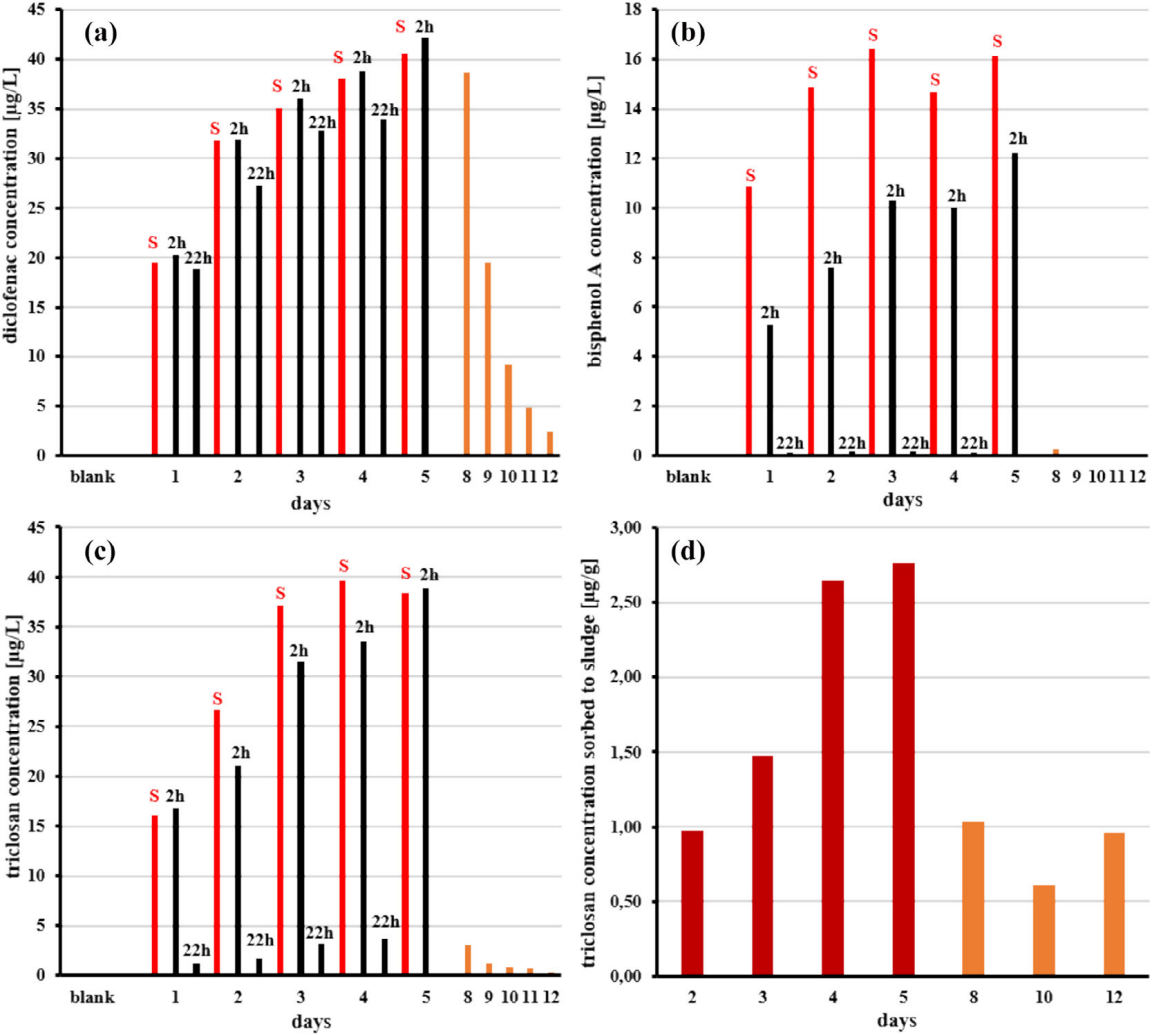
Micropollutant concentrations during SBR treatment; feed graywater spiked with micropollutants was exchanged daily (red bars [S]) indicate samples collected right after graywater addition; black bars (2, 22 h) represent samples collected 2 and 22 h thereafter; orange bars show daily samples collected after micropollutants application to the synthetic graywater; (a) diclofenac concentration in SBR; (b) bisphenol A concentration in SBR; (c) triclosan concentration in SBR; (d) triclosan concentration extracted from the sludge.

The investigated micropollutants are structurally very variable, and, hence, their biodegradability and stability against abiotic degradation differs widely. Nevertheless, regarding the behavior during the SBR treatment, the respective compounds can be roughly assigned to a few groups with similar characteristics. Figure 2 gives a representative overview of the time‐dependent behavior of those micropollutant groups.

A first group is rather stable against the SBR treatment. Only minor removal can be observed within 22 h, and therefore the substances are accumulating in the bioreactor over the week. This in turn leads to rising concentrations in the SBR (see Figure 2a diclofenac). In the week after, when graywater without supplemented micropollutants is used for feeding, the concentration decreases due to dilution by daily exchange with micropollutant‐free synthetic graywater. Besides diclofenac, the insect repellent DEET also belongs to this group. 1H‐benzotriazole and terbutryn show slightly better but still incomplete removal rates within 22 h of 65% and 30%, respectively.

The insufficient elimination of the four substances diclofenac, DEET, 1H‐benzotriazole, and terbutryn by aerobic biological treatment is confirmed by studies of other authors. For example, it is known that the aerobic biological stage of wastewater treatment is insufficiently efficient with regard to diclofenac (e.g., Butkovskyi et al., 2015). Liu et al. (2023) showed by comparing the influent and effluent of a WWTP that the concentration of DEET is significantly reduced, but only about 50% of the DEET is eliminated in the aerated sand chamber as the first stage, mainly by sorption to particles. In contrast, the sorption of DEET to activated sludge was of little importance in our own investigations. In Liu's study, virtually complete elimination only occurred in a second stage (anaerobic and anoxic treatment, secondary sedimentation). The removability of DEET in different WWTPs varies greatly (10% to 90%), which is explained by different boundary conditions such as technical processes, plant size, dilution by rain, microbiological community, or temperature (Stalder et al., 2022). The removability of terbutryn in WWTPs and in laboratory tests has also been reported as incomplete (Chand et al., 2017; Luft et al., 2014). For 1H‐benzotriazole, both aerobic and anaerobic laboratory tests have shown that the substance is only slowly degraded (Liu et al., 2011).

Bisphenol A (Figure 2b), octinoxate, and the parabens belong to a second group and are removed rapidly, thereby avoiding accumulation in the SBR. The results regarding the biodegradability of parabens are in good agreement with the literature. Both field studies and model simulations by Zhang et al. (2021) showed that various parabens are largely removed by biodegradation in WWTPs. The same authors were also able to demonstrate that the good elimination of triclocarban and triclosan (see next section on the third group) is primarily based on sorption on activated sludge. The efficient removal of bisphenol A and parabens by biological treatment has also been proven in real graywater and was explained by a combination of their high degradability under aerobic conditions and adsorption on sewage sludge (Hernández Leal, Vieno, et al., 2010). In contrast, the same authors found rather low octinoxate removal in real graywater during biological treatment, what is rather surprising concerning the strong theoretical sorption tendency of this hydrophobic UV filter to activated sludge (see Table 3). But on the other hand, in the present study, no significant enrichment in corresponding sludge samples has been detected. Therefore, the difference in the removal rates cannot be conclusively clarified.

The third group comprises very hydrophobic compounds such as triclosan (Figure 2c), triclocarban, and chlorpyrifos, which are removed rapidly from the water body but accumulate in the sludge in agreement with the estimate based on corresponding log K_OC_ values specified in Table 3 (Figure 2). The high sorptivity of triclosan and triclocarban to activated sludge has already been reported (Wick et al., 2011). Concerning the reuse of the treated water itself, the sorption is not a major problem, but usage of the sludge, for example, as biosolids for agricultural purposes, must be considered with care, since it has been reported, that uptake of triclosan and triclocarban from biosolids into plant roots (e.g., soybean) is possible. However, the compounds are less available from biosolids than from contaminated irrigation water (Wu et al., 2010). Both compounds are moderately biodegradable in soil under aerobic conditions (Ying et al., 2007), with half‐lives of 20–58 days (triclosan) and 87–231 days (triclocarban) in biosolids‐amended soils (Wu et al., 2009). The problem has also been reported for biosolids from municipal WWTPs, where even with conventional sludge processing systems, a 90% reduction of triclocarban or triclosan from the biosolids seems not be practicable (Ogunyoku & Young, 2014).

Table 4 summarizes the achieved micropollutant removal during SBR treatment and compares the obtained values to corresponding Environmental Quality Standard (EQS) values. Only in the case of the parabens and 1H‐benzotriazol the SBR treatment is sufficient to comply with EQS values. The elimination of DEET is poor but under the selected conditions sufficiently good to reach the EQS values. All the other substances need a further treatment step. The comparatively high concentrations of triclosan, triclocarban, and chlorpyrifos in the sludge (2.8, 1.9, and 2.1 μg/g, respectively) after 5 days confirm their classification above into the third group of hydrophobic micropollutants that accumulate well on the sludge.

**TABLE 4:** Micropollutant removal by sequencing batch reactor (SBR) treatment.

Micropollutant	β_GW_ [μg/L]	β_SBR,1d_^in^ [μg/L]	Removal within 24 h*	β_SBR,5d_^in^ [μg/L]	w_sludge,5d_ [μg/g]	β_SBR,24_^out^ [μg/L]	[μg/L]
Methylparaben	43.1 ± 2.7	0.8	(66 ± 44) %	2.7	0.02	0.79	1.3^#, (a)^
Benzylparaben	48.1 ± 3.0	0.4	>95%	1.2	<0.01	<0.02	0.04^#, (a)^
Triclosan	31.7 ± 2.1	16.1	(92.1 ± 1.4) %	38.4	2.8	3.8	0.02^(b)^
Triclocarban	18.3 ± 2.9	2.8	(91.4 ± 9.6) %	29.8	1.9	0.63	0.094^#, (c)^
Octinoxate	23.6 ± 3.1	7.6	(98.8 ± 0.7) %	7.2	0.03	<0.15	0.02^#, (d)^
1H‐benzotriazol	36.7 ± 2.9	24.0	(65.1 ± 5.3) %	30.9	0.02	10.7	19^(e)^
Bisphenol A	43.4 ± 3.3	10.8	(98.9 ± 0.2) %	16.1	0.02	<0.25	0.1^(f)^
Diclofenac	40.3 ± 4.1	19.6	(8.7 ± 4.7) %	40.5	0.1	33.9	0.05^(e)^
DEET	37.1 ± 2.6	19.3	(4.1 ± 4.1) %	39.6	0.08	33.2	71^(f)^
Chlorpyrifos	15.9 ± 1.5	2.7	(85.0 ± 4.3) %	6.4	2.1	0.99	0.03^(g)^
Terbutryn	13.0 ± 0.5	9.4	(29.8 ± 6.5) %	15.1	0.4	10.1	0.065^(g)^

*Note*: β_GW_ determined mass concentration in applied graywater (*n* = 5), β_SBR,1d_^in^ and β_SBR,5d_^in^ determined mass concentrations at first and fifth day of the experiment just after graywater addition, β_SBR,22h_^out^ maximum mass concentrations after 24 h of SBR treatment, w_sludge,5d_ determined mass fraction in sludge at the fifth day of the experiment, *average elimination within 24 h over five consecutive days ± standard deviation; ^#^Predicted No Effect Concentrations (PNEC) instead of Environmental Quality Standard (EQS) reported, ^(a)^ according to Terasaki et al., 2015, ^(b)^ according to German OgewV,(OGewV, 2016) ^(c)^ according to ECHA database, ^(d)^ according to Ma et al., 2016, ^(e)^ recommendation of Swiss Centre for Applied Ecotoxicology, ^(f)^ recommendation of German LAWA, ^(g)^ according to Directive 2013/39/EU (EC, 2000).

AOP using a combination of H_2_O_2_ and UV is a very powerful approach for an efficient elimination of micropollutants in pretreated wastewater. By producing hydroxyl radicals, AOP rather nonspecifically degrades organic compounds. This means that the graywater composition has a huge impact on the elimination efficiency. Thus, the AOP treatment decreases the concentrations of each of the studied micropollutants, however, with varying efficiency.

**FIGURE 3:**
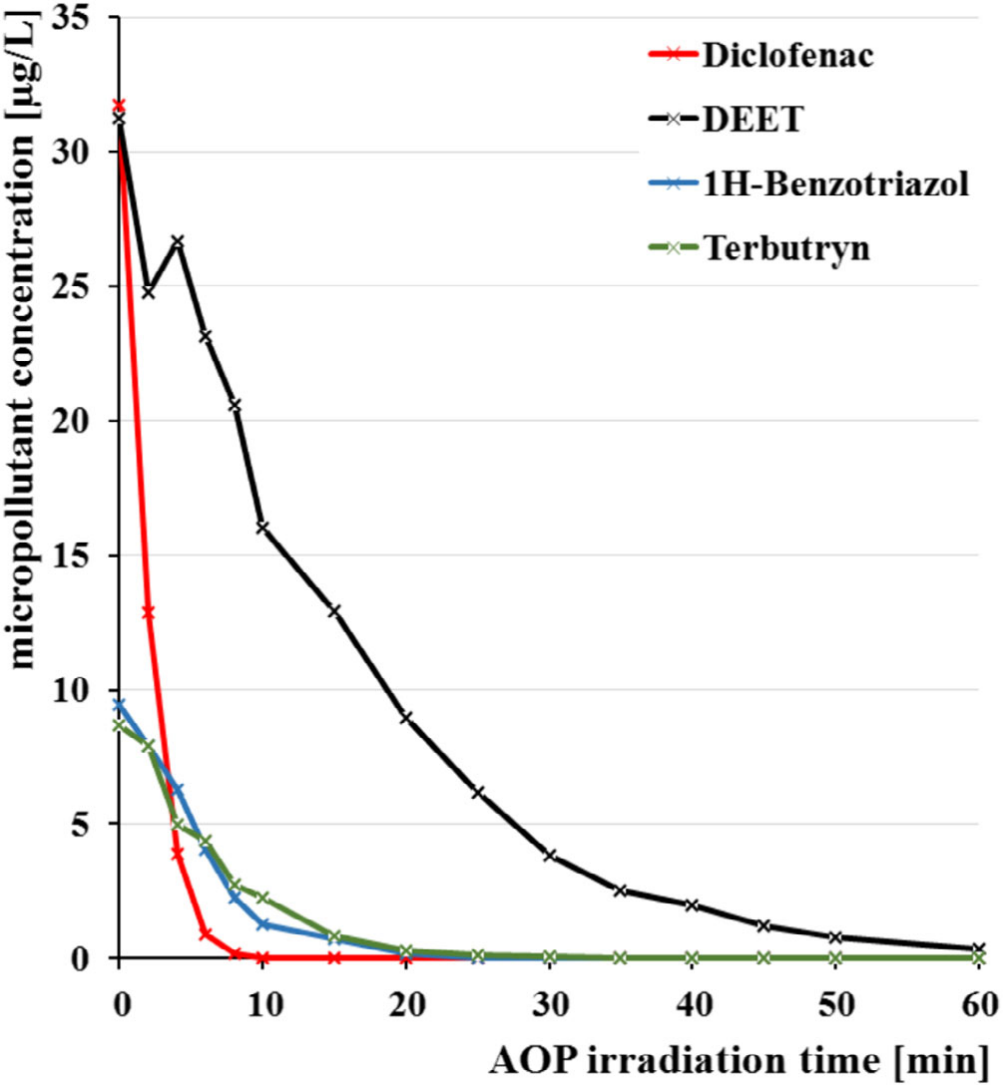
Micropollutant removal by subsequent UV/H_2_O_2_ advanced oxidation processes (AOP) treatment of SBR effluent.

Figure 3 shows the time‐dependent degradation curves for four selected micropollutants during AOP treatment, which showed no/only slow elimination during the previous SBR treatment of the graywater (Figure 3, Group A). It is apparent, that diclofenac is much easier to degrade than DEET. But since the EQS for DEET is by far higher than for diclofenac, the concentration limits to be met can still be reached.

The left columns of Table 5 summarize the values for the time required for 90% elimination and for the corresponding energy efficiency of the AOP treatment for each micropollutant, based on experiments with SBR treated graywater. Here, the graywater was subsequently spiked with micropollutants just before the AOP treatment step in order to ensure sufficient micropollutant availability even for well‐biodegradable compounds. In contrast, the right part of Table 5 lists determined micropollutant concentrations of graywater, which has been consecutively treated by SBR and subsequently by AOP for 10, 20, and 60 min, respectively. After 20 min of irradiation, all EQS values are reached except for terbutryn, which would need a treatment time of >60 min to achieve EQS compliance. The calculated electrical energy per order (EE/O) values are relatively high compared to literature (e.g., terbutryn 15.8 vs. 0.95 kWh/m^3^) (James et al., 2014), triclosan 4.08 vs. 1.6 kWh/m^3^ (without H_2_O_2_ addition; Dubowski et al. (2020)). The SBR effluent has only been coarsely filtrated prior to AOP treatment, thus, the remaining light scattering particles forestall higher energy efficiency. Moreover, inorganic salts and dissolved organic matter might act as radical scavenger and reduce AOP efficiency compared to results obtained in distilled water or similarly low polluted media (Ulliman et al., 2018).

**TABLE 5:** Micropollutant removal by subsequent advanced oxidation processes (AOP) treatment.

Micropollutant	Treatment time for 90% elimination^†^ [min]	EE/O^†^ [kWh/m^3^]	Treatment time for EQS compliance† [min]	β_10min_* (max) [μg/L]	β_20min_* (max) [μg/L]	β_60min_* (max) [μg/L]
Methylparaben	28.8	24.0	SBR sufficient	0.83	0.77	0.64
Benzylparaben	18.4	15.3	SBR sufficient	< 0.02	< 0.02	< 0.02
Triclosan	4.9	4.08	11.2	0.04	< 0.02	< 0.02
Triclocarban	17.0	14.2	14.0	< 0.15	< 0.15	< 0.15
Octinoxate	4.9	4.08	4.3^#^	<0.15	< 0.15	< 0.15
1H‐benzotriazol	5.8	4.83	SBR sufficient	0.9	< 0.02	< 0.02
Bisphenol A	13.5	11.2	5.4^#^	< 0.25	< 0.25	< 0.25
Diclofenac	4.6	3.83	13.0	< 0.02	< 0.02	< 0.02
DEET	27.8	23.2	SBR sufficient	16	< 0.02	< 0.02
Chlorpyrifos	26.5	22.1	40.2	0.03	< 0.02	< 0.02
Terbutryn	19.0	15.8	>60	3.8	1.7	0.10

*Note*: EE/O = electrical energy per order, ^†^values were determined in a separate experiment using SBR effluent, which was spiked with micropollutants; * maximum mass concentrations within the experiment period of 5 days after 10, 20, and 60 min AOP treatment.

^#^ effluent concentration of SBR was set to LOQ for calculation as worst case scenario.

Abbreviation: EQS, Environmental Quality Standard; SBR, sequencing batch reactor.

### Compliance of the reclaimed water with reuse standards

The achieved water quality determines the possible reuse options. Table 6 compares the effluent values of different parameters obtained during the subsequent SBR and AOP treatment steps with selected Vietnamese regulations and somewhat stricter regulations from an EU proposal and US EPA (the latter one additionally requires residual chlorine in the reused water, which would make a chlorination step necessary).

**TABLE 6:** Achieved water quality in comparison to relevant regulations.

Parameter	Studied model graywater				
	Before treatment	After SBR treatment	After SBR + Filtration/AOP (20 min)	Comparison to selected regulations	
**Total coliforms** **[MPN/100 mL]**	>2400	1320	<1	QCVN 392011, Vietnam^(1)^	Fecal: <200
				TCVN 6772:2000, Vietnam^(2)^	<1000
				TCVN 5945:2005, Vietnam^(3)^	<3000
				EU regulation^(4)^	<10
				USEPA Guidelines^(5)^	<2.2
**Turbidity [NTU]**	158	8	0.7–1.2	QCVN 392011, Vietnam^(1)^	‐
				TCVN 6772:2000, Vietnam^(2)^	‐
				TCVN 5945:2005, Vietnam^(3)^	‐
				EU regulation^(4)^	<5
				USEPA Guidelines^(5)^	<2
**pH**	8.4	7.7–8.6	8.7	QCVN 392011, Vietnam^(1)^	5.5–9.0
				TCVN 6772:2000, Vietnam^(2)^	5.0–9.0
				TCVN 5945:2005, Vietnam^(3)^	6.0–9.0
				EU regulation^(4)^	‐
				USEPA Guidelines^(5)^	6.0–9.0
**Pt/co**	91–126	21–26	4–8	QCVN 392011, Vietnam^(1)^	‐
				TCVN 6772:2000, Vietnam^(2)^	‐
				TCVN 5945:2005, Vietnam^(3)^	<20
				EU regulation^(4)^	‐
				USEPA Guidelines^(5)^	‐
**BOD5** **[mg/L]**	130–210	5–6	2–4	QCVN 392011, Vietnam^(1)^	‐
				TCVN 6772:2000, Vietnam^(2)^	<30
				TCVN 5945:2005, Vietnam^(3)^	<30
				EU regulation^(4)^	<10
				USEPA Guidelines^(5)^	<10
**DO** **[mg/L]**	>3	>3	‐	QCVN 392011, Vietnam^(1)^	>2
				TCVN 6772:2000, Vietnam^(2)^	‐
				TCVN 5945:2005, Vietnam^(3)^	‐
				EU regulation^(4)^	‐
				USEPA Guidelines^(5)^	‐
**N‐species** **[mg/L]**	NO_3_^**−**^ **‐N:** 4–6.6 **NH**_**4**_^**+**^ **‐N:** 29–37 TN: 50–52	**NO**_**3**_^**−**^ **‐N:** 20–42 **NH**_**4**_^**+**^ **‐N:** 0.03–0.07	**TN:** 30–33	QCVN 392011, Vietnam^(1)^	‐
				TCVN 6772:2000, Vietnam^(2)^	NO_3_^−^‐N: <30
				TCVN 5945:2005, Vietnam^(3)^	NH_4_^+^‐N: <5
				EU regulation^(4)^	‐
				USEPA Guidelines^(5)^	‐
**P species [mg/L]**	TP: 6.9–7.5	TP: 4.1–6.2	‐	QCVN 392011, Vietnam^(1)^	‐
				TCVN 6772:2000, Vietnam^(2)^	PO_4_^3−^: <6
				TCVN 5945:2005, Vietnam^(3)^	TP: <4
				EU regulation^(4)^	‐
				USEPA Guidelines^(5)^	‐

*Note*: ^(1)^ QCVN 39:2011/BTNMT, Vietnam (Water Quality Standards for irrigated agriculture) (QCVN, 2011); ^(2)^ TCVN 6772:2000, Vietnam (domestic wastewater discharge; disposing site Level I) (TCVN, 2000); ^(3)^ TCVN 5945:2005, Vietnam (industrial wastewater discharge; A: receiving water as supply for domestic reuse) (TCVN, 2005); ^(4)^ Proposal for a Regulation of the European Parliament and of the Council on minimum requirements for water reuse, 2018/0169 (chemical oxygen demand [COD]) (agriculture for food production) (European Commission, 2018); ^(5)^ *Guidelines for Water Reuse*, US EPA, 2012, EPA/600/R‐12/618.

Abbreviations: AOP, advanced oxidation processes; DO, dissolved oxygen; SBR, sequencing batch reactor; TP, total phosphorus.

As expected, still rather high values were determined for coliforms after SBR treatment. Only filtration/AOP resulted in a considerable reduction by more than three orders of magnitude, thus fulfilling all requirements of the specified regulations. The turbidity also remains at a very high level after the biological stage and was only sufficiently removed by the posttreatment. The pH range is maintained within the required range during the whole treatment. Regarding discoloration, it is considered essential that the requirements for “Water Quality Standards for irrigated agriculture” as well as USEPA Guidelines for “Water reuse” are achieved with advanced graywater treatment, since the objective of the study was to reuse the treated water appropriately (e.g., irrigation, toilet, and groundwater recharge). Regarding better elimination of P‐ and N‐species, biological treatment needs to be further optimized, as no significant removal in this regard is expected by AOP posttreatment. Overall, it can be concluded that the main objectives of the present study can be reached by the studied process combination.

The SBR treatment step is sufficient to comply with the requirements set for nutrient removal in most regulations. The major problem is the insufficient removal of coliforms and color by mere biological treatment. Furthermore, turbidity and, as mentioned above, most of the micropollutants are not eliminated efficiently. A more effective removal of suspended solids and the associated significant increase in energy efficiency in the subsequent AOP can be achieved by efficient filtration, for example, by simple sand filters or by applying membrane bioreactors, which are already increasingly used in Vietnam, instead of SBR. A subsequent AOP treatment is well‐suited to remedy the shortcomings of the SBR in terms of micropollutant removal. The SBR/AOP combination affords therefore a sufficient water quality for domestic or agricultural use or direct discharge into adjacent receiving waters.

Before transferring the proposed process combination into practice, further investigations may have to be carried out with regard to the occurrence of other regionally specific micropollutants in the respective graywater and their removability by a subsequent AOP treatment step. Furthermore, the corresponding costs of the treatment technique (investment and operation) should be compared with the respective costs for processed water and alternative graywater posttreatment approaches such as constructed wetlands, which, however, was not the subject of the present preliminary study on the general suitability of the process combination.

## CONCLUSION

Graywater can be reused for various applications after appropriate treatment, which is particularly significant for countries with projected increasing problems regarding water availability and quality, such as Vietnam. However, a solely biological treatment is mostly insufficient. A subsequent UV/peroxide treatment leads to a significant improvement of various general water quality parameters and reduction of micropollutant concentrations.

The key findings are as follows:

- Since graywater is very heterogeneous and of fluctuating quality, a synthetically produced graywater could be successfully generated and used for the laboratory tests. The model graywater remained sufficiently stable during the period of tests with regard to the studied parameters.
- During the aerobic biological treatment of the synthetic graywater using an SBR, the water quality could be significantly improved with respect to the parameters of SAC‐436, COD, BOD5, and ammonium. However, as expected, the aerobic biological stage is not sufficient to reduce turbidity, coliforms, total P, and total N.
- The studied micropollutants could be classified into a group that is non/barely removable, a group that is microbially degradable, and a group that is eliminable by sorption during SBR treatment. However, only three compounds could be removed to such an extent by SBR treatment alone that their concentration reached the set EQS.
- By means of a subsequent advanced treatment using an AOP process (VUV and peroxide), the micropollutant concentrations could be reduced below the respective EQS except for terbutryn.
- Filtration and the AOP process were also able to bring SAC‐436, turbidity, and coliforms into a range complying with the relevant guidelines and regulations.

## AUTHOR CONTRIBUTIONS

**Stephan Beil:** Writing—original draft; conceptualization; validation; investigation. **Amélie Chabilan:** Investigation; data curation. **Linda Schuster:** Conceptualization; data curation; methodology; investigation. **Hilmar Börnick:** Supervision; funding acquisition; writing—review and editing. **Minh Tan Nguyen:** Writing—review and editing; funding acquisition. **Stefan Stolte:** Conceptualization; supervision; writing—review and editing; methodology.

## CONFLICT OF INTEREST STATEMENT

The authors declare no conflict of interests.

## Supporting information


**Table S1.** List of chemicals, standards and internal isotopically labeled standards ISTD with their manufacturers or supplier.
**Figure S1:** Image of the SBR in the aeration/agitation phase (left) and settling phase (right).
**Figure S2:** Experimental setup with UV reactor.
**Table S2.** MRMs and retention times for LC–MS/MS analyses of micropollutants.

## Data Availability

The data that support the findings of this study are available from the corresponding author upon reasonable request.

## References

[wer70096-bib-0001] Abed, S. N. , & Scholz, M. (2016). Chemical simulation of greywater. Environmental Technology, 37(13), 1631–1646. 10.1080/09593330.2015.1123301 26745659

[wer70096-bib-0002] Almqvist, H. , & Hanæus, J. (2006). Organic hazardous substances in graywater from Swedish households. Journal of Environmental Engineering, 132(8), 901–908. 10.1061/(ASCE)0733-9372(2006)132:8(901)

[wer70096-bib-0003] Asano, T. , Burton, F. , Leverenz, H. , Tsuchihashi, R. , & Tchobanoglous, G. (2007). Water reuse: Issues, technologies, and applications. Metcalf & Eddy. ISBN13: 9780

[wer70096-bib-0004] ATV‐DVWK . (1997). Biologische und weitergehende Abwasserreinigung. Ernst & Sohn Verlag für Architektur und technische Wissenschaften GmbH. ISBN13: 9783433014622.

[wer70096-bib-0005] Balmer, M. E. , Buser, H.‐R. , Müller, M. D. , & Poiger, T. (2005). Occurrence of some organic UV filters in wastewater, in surface waters, and in fish from Swiss Lakes. Environmental Science & Technology, 39(4), 953–962. 10.1021/es040055r 15773466

[wer70096-bib-0006] Boyjoo, Y. , Pareek, V. K. , & Ang, M. (2013). A review of greywater characteristics and treatment processes. Water Science and Technology, 67(7), 1403–1424. 10.2166/wst.2013.675 23552228

[wer70096-bib-0007] Büsser, S. , Pham, T. N. , Morel, A. , & Nguyen, V. A. (2007). Characteristics and quantities of domestic wastewater in urban and peri‐urban households in Hanoi. In Annual report of FY 2006, the Core University program between Japan Society for the Promotion of Science (JSPS) and Vietnamese academy of science and technology (VAST) (pp. 395–397).

[wer70096-bib-0008] Butkovskyi, A. , Hernandez Leal, L. , Rijnaarts, H. H. M. , & Zeeman, G. (2015). Fate of pharmaceuticals in full‐scale source separated sanitation system. Water Research, 85, 384–392. 10.1016/j.watres.2015.08.045 26364222

[wer70096-bib-0009] Chand, R. , Tulucan, T. , & Aburlacitei, M. (2017). Investigation of biocide biodegradation in wastewater under laboratory set‐up in anaerobic, aerobic and aerobic with substrate conditions. Journal of Civil & Environmental Engineering, 08(01). 10.4172/2165-784X.1000295

[wer70096-bib-0010] COSMOtherm, version C30, release 18; COSMOlogic GmbH & Co. KG; http://www.cosmologic.de. (2018).

[wer70096-bib-0011] Deshayes, S. , Eudes, V. , Droguet, C. , Bigourie, M. , Gasperi, J. , & Moilleron, R. (2015). Alkylphenols and phthalates in greywater from showers and washing machines. Water, Air, and Soil Pollution, 226(11), 388. 10.1007/s11270-015-2652-7

[wer70096-bib-0012] DIN 38404–3:2005–07 (2005). German standard methods for the examination of water, waste water and sludge—Physical and physical‐chemical parameters (group C)—Part 3: Determination of absorption in the range of the ultraviolet radiation, spectral absorptions c. German Institute for Standardization.

[wer70096-bib-0013] DIN 38405–9:2011–09 (2011). German standard methods for examination of water, waste water and sludge—Anions (group D)—Part 9: Spectrometric determination of nitrate (D 9). German Institute for Standardization.

[wer70096-bib-0014] DIN 38406–5:1983–10 (1983). German standard methods for the examination of water, waste water and sludge; cations (group E); determination of ammonia‐nitrogen (E 5). German Institute for Standardization.

[wer70096-bib-0015] DIN 38409–1:1987–01 (1987). German standard methods for the examination of water, waste water and sludge; parameters characterizing effects and substances (group H); determination of total dry residue, filtrate dry residue and residue on ignition (H 1). German Institute for Standardization.

[wer70096-bib-0016] DIN 38414–10:1981–09 (1981). German standard methods for the analysis of water, waste water and sludge; sludge and sediments (group S); determination of the proportion of sludge volume and sludge volume index (S 10). German Institute for Standardization.

[wer70096-bib-0017] DIN EN 12260:2003–12 (2003). Water quality—Determination of nitrogen—Determination of bound nitrogen (TNb), following oxidation to nitrogen oxides; German version EN 12260:2003. German Institute for Standardization.

[wer70096-bib-0018] DIN EN 1484:2019‐04 (2019). Water analysis—Guidelines for the determination of total organic carbon (TOC) and dissolved organic carbon (DOC); German version EN 1484:1997. German Institute for Standardization.

[wer70096-bib-0019] DIN EN 1899‐1:1998‐05 (1998). Water quality—Determination of biochemical oxygen demand after n days (BOD_n_)—Part 1: Dilution and seeding method with allylthiourea acid addition (ISO 5815:1989, modified); German version EN 1899‐1:1998. German Institute for Standardization.

[wer70096-bib-0020] DIN EN ISO 6271:2016–05 (2016). Clear liquids—Estimation of colour by the platinum‐cobalt colour scale (ISO 6271:2015); German version EN ISO 6271:2015. German Institute for Standardization.

[wer70096-bib-0021] DIN EN ISO 6878:2004‐09 (2004). Water quality—Determination of phosphorus—Ammonium molybdate spectrometric method (ISO 6878:2004); German version EN ISO 6878:2004. German Institute for Standardization.

[wer70096-bib-0022] DIN EN ISO 7887:2012‐04 (2012). Water quality—Examination and determination of colour (ISO 7887:2011); German version EN ISO 7887:2011. German Institute for Standardization.

[wer70096-bib-0023] DIN ISO 15705:2003–01 (2003). Water quality—Determination of the chemical oxygen demand index (ST‐COD)—Small‐scale sealed tube method (ISO 15705:2002). German Institute for Standardization.

[wer70096-bib-0024] Dubowski, Y. , Alfiya, Y. , Gilboa, Y. , Sabach, S. , & Friedler, E. (2020). Removal of organic micropollutants from biologically treated greywater using continuous‐flow vacuum‐UV/UVC photo‐reactor. Environmental Science and Pollution Research, 27(7), 7578–7587. 10.1007/s11356-019-07399-7 31885065

[wer70096-bib-0025] EC . (2000). Directive 2000/60/EC of the European Parliament and of the Council of 23 October 2000 establishing a framework for community action in the field of water policy. Official Journal of the European Parliament L 327.

[wer70096-bib-0026] Eckert, F. , & Klamt, A. (2002). Fast solvent screening via quantum chemistry: COSMO‐RS approach. AICHE Journal, 48, 369–385. 10.1002/aic.690480220

[wer70096-bib-0027] Eriksson, E. , Andersen, H. R. , Madsen, T. S. , & Ledin, A. (2009). Grey water pollution variability and loadings. Ecological Engineering, 35(5), 661–669. 10.1016/j.ecoleng.2008.10.015

[wer70096-bib-0028] Famiglietti, J. S. (2014). The global groundwater crisis. Nature Climate Change, 4(11), 945–948. 10.1038/nclimate2425

[wer70096-bib-0029] Filali, H. , Barsan, N. , Souguir, D. , Nedeff, V. , Tomozei, C. , & Hachicha, M. (2022). Greywater as an alternative solution for a sustainable Management of Water Resources—A Review. Sustainability, 14(2), 665. 10.3390/su14020665

[wer70096-bib-0030] Geiling E.‐L. (2015). Removal of the micropollutants DEET and DEP in biological grey water treatment and the effect of DEP on microbiological processes; Norwegian University of Science and Technology—Trondheim

[wer70096-bib-0031] Ghaitidak, D. M. , & Yadav, K. D. (2013). Characteristics and treatment of greywater—A review. Environmental Science and Pollution Research, 20(5), 2795–2809. 10.1007/s11356-013-1533-0 23397178

[wer70096-bib-0032] US EPA (2012). Guidelines for water reuse. U. S. Environmental Protection Agency. EPA/600/R‐12/618. (n.d.)

[wer70096-bib-0033] Heidler, J. , Sapkota, A. , & Halden, R. U. (2006). Partitioning, persistence, and accumulation in digested sludge of the topical antiseptic triclocarban during wastewater treatment. Environmental Science & Technology, 40(11), 3634–3639. 10.1021/es052245n 16786704 PMC2768036

[wer70096-bib-0034] Hernández Leal, L. , Temmink, H. , Zeeman, G. , & Buisman, C. J. N. (2010). Comparison of three systems for biological greywater treatment. Water (Basel), 2(2), 155–169.

[wer70096-bib-0035] Hernández Leal, L. , Vieno, N. , Temmink, H. , Zeeman, G. , & Buisman, C. J. N. (2010). Occurrence of xenobiotics in gray water and removal in three biological treatment systems. Environmental Science & Technology, 44(17), 6835–6842. 10.1021/es101509e 20681737

[wer70096-bib-0036] Hernández‐Leal, L. , Temmink, H. , Zeeman, G. , & Buisman, C. J. N. (2011). Removal of micropollutants from aerobically treated grey water via ozone and activated carbon. Water Research, 45(9), 2887–2896. 10.1016/j.watres.2011.03.009 21453950

[wer70096-bib-0037] Hiwat, H. , Doerdjan, K. , Kerpens, M. , Samjhawan, A. , & Soekhoe, T. (2013). Importance of domestic water containers as *Aedes aegypti* breeding sites in Suriname; implications for dengue control. Academic Journal of Suriname 4/1, 4403–4407.

[wer70096-bib-0038] Hourlier, F. , Masse, A. , Jaouen, P. , Lakel, A. , Gerente, C. , Faur, C. , & Le Cloirec, P. (2010). Formulation of synthetic greywater as an evaluation tool for wastewater recycling technologies. Environmental Technology, 31(2), 215–223. 10.1080/09593330903431547 20391806

[wer70096-bib-0039] James, C. P. , Germain, E. , & Judd, S. (2014). Micropollutant removal by advanced oxidation of microfiltered secondary effluent for water reuse. Separation and Purification Technology, 127, 77–83. 10.1016/j.seppur.2014.02.016

[wer70096-bib-0040] Jensen, O. , & Yu, X. (2016). Wastewater reuse in Beijing: An evolving hybrid system. International Journal of Water Resources Development, 32(4), 590–610. 10.1080/07900627.2016.1148589

[wer70096-bib-0041] Jimenez, B. , & Asano, T. (2008). Water reuse: An international survey of current practice, issues and needs. IWA Publishing.

[wer70096-bib-0042] Klamt, A. , & Eckert, F. (2000). COSMO‐RS: A novel and efficient method for the a priori prediction of thermophysical data of liquids. Fluid Phase Equilibria, 172(1), 43–72. 10.1016/S0378-3812(00)00357-5

[wer70096-bib-0043] Klamt, A. , Eckert, F. , & Diedenhofen, M. (2002). Prediction of soil sorption coefficients with a conductor‐like screening model for real solvents. Environmental Toxicology and Chemistry, 21, 2562–2566. 10.1002/etc.5620211206 12463549

[wer70096-bib-0044] Klamt, A. , Eckert, F. , Hornig, M. , Beck, M. E. , & Bürger, T. (2002). Prediction of aqueous solubility of drugs and pesticides with COSMO‐RS. Journal of Computational Chemistry, 23(2), 275–281. 10.1002/jcc.1168 11924739

[wer70096-bib-0045] Lamine, M. , Bousselmi, L. , & Ghrabi, A. (2007). Biological treatment of grey water using sequencing batch reactor. Desalination, 215(1–3), 127–132. 10.1016/j.desal.2006.11.017

[wer70096-bib-0046] Langenhoff, A. , Inderfurth, N. , Veuskens, T. , Schraa, G. , Blokland, M. , Kujawa‐Roeleveld, K. , & Rijnaarts, H. (2013). Microbial removal of the pharmaceutical compounds ibuprofen and diclofenac from wastewater. BioMed Research International, 2013, 325806. 10.1155/2013/325806 24350260 PMC3852090

[wer70096-bib-0047] Le, V. H. , Pham, Q. N. , Phung, T. L. , Doan, V. C. , Tran, Q. C. , & Dang, T. T. (2024). The role of groundwater recharge in groundwater exploitation of the red river delta plain. Ministry of Science and Technology, Vietnam, 66(3), 120–128.

[wer70096-bib-0048] Liu, J. , Ge, S. , Shao, P. , Wang, J. , Liu, Y. , Wei, W. , He, C. , & Zhang, L. (2023). Occurrence and removal rate of typical pharmaceuticals and personal care products (PPCPs) in an urban wastewater treatment plant in Beijing, China. Chemosphere, 339, 139644. 10.1016/j.chemosphere.2023.139644 37495050

[wer70096-bib-0049] Liu, Y.‐S. , Ying, G.‐G. , Shareef, A. , & Kookana, R. S. (2011). Biodegradation of three selected benzotriazoles under aerobic and anaerobic conditions. Water Research, 45(16), 5005–5014. 10.1016/j.watres.2011.07.001 21802111

[wer70096-bib-0050] Loschen C. Hellweg A. Klamt A. 2016 COSMOquick, version 1.5; COSMOlogic GmbH & co. KG, Leverkusen, Germany

[wer70096-bib-0051] Loschen, C. , & Klamt, A. (2012). COSMOquick : A novel Interface for fast σ‐profile composition and its application to COSMO‐RS solvent screening using multiple reference solvents. Industrial and Engineering Chemistry Research, 51(43), 14303–14308. 10.1021/ie3023675

[wer70096-bib-0052] Luft, A. , Wagner, M. , & Ternes, T. A. (2014). Transformation of biocides Irgarol and terbutryn in the biological wastewater treatment. Environmental Science & Technology, 48(1), 244–254. 10.1021/es403531d 24328195

[wer70096-bib-0053] Ma, B. , Lu, G. , Liu, F. , Nie, Y. , Zhang, Z. , & Li, Y. (2016). Organic UV filters in the surface water of Nanjing, China: Occurrence, distribution and ecological risk assessment. Bulletin of Environmental Contamination and Toxicology, 96(4), 530–535. 10.1007/s00128-015-1725-z 26747437

[wer70096-bib-0054] Marino, D. , & Ronco, A. (2005). Cypermethrin and chlorpyrifos concentration levels in surface water bodies of the Pampa Ondulada, Argentina. Bulletin of Environmental Contamination and Toxicology, 75(4), 820–826. 16400566 10.1007/s00128-005-0824-7

[wer70096-bib-0055] Montangero, A. , Cau, L. N. , Anh, N. V. , Tuan, V. D. , Nga, P. T. , & Belevi, H. (2007). Optimising water and phosphorus management in the urban environmental sanitation system of Hanoi, Vietnam. Science of the Total Environment, 384(1–3), 55–66. 17604824 10.1016/j.scitotenv.2007.05.032

[wer70096-bib-0056] Neitzel, V. , & Iske, U. (1998). Abwasser, Technik und Kontrolle. Wiley‐VCH Verlag GmbH & Co. KGaA.

[wer70096-bib-0057] Nghiem, L. D. , Oschmann, N. , & Schäfer, A. I. (2006). Fouling in greywater recycling by direct ultrafiltration. Desalination, 187(1–3), 283–290. 10.1016/j.desal.2005.04.087

[wer70096-bib-0058] Nguyen, T. T. , Kawamura, A. , Tong, T. N. , Nakagawa, N. , Amaguchi, H. , & Gilbuena, R. (2014). Hydrogeochemical characteristics of groundwater from the two main aquifers in the Red River Delta, Vietnam. Journal of Asian Earth Sciences, 93, 180–192.

[wer70096-bib-0059] Noh, S. , Kwon, I. , Yang, H.‐M. , Choi, H.‐L. , & Kim, H. (2004). Current status of water reuse systems in Korea. Water Science and Technology, 50(2), 309–314. 10.2166/wst.2004.0146 15344806

[wer70096-bib-0060] Noutsopoulos, C. , Andreadakis, A. , Kouris, N. , Charchousi, D. , Mendrinou, P. , Galani, A. , Mantziaras, I. , & Koumaki, E. (2018). Greywater characterization and loadings—Physicochemical treatment to promote onsite reuse. Journal of Environmental Management, 216, 337–346. 10.1016/j.jenvman.2017.05.094 28592390

[wer70096-bib-0061] OGewV (2016). Oberflächengewässerverordnung vom 20. Juni 2016 (BGBl. I S. 1373), die zuletzt durch Artikel 2 Absatz 4 des Gesetzes vom 9. Dezember 2020 (BGBl. I S. 2873) geändert worden ist. Bundesministeriums der Justiz sowie des Bundesamts für Justiz.

[wer70096-bib-0062] Ogunyoku, T. A. , & Young, T. M. (2014). Removal of Triclocarban and Triclosan during municipal biosolid production. Water Environment Research, 86(3), 197–203. 10.2175/106143013X13807328849378 24734467 PMC3989550

[wer70096-bib-0063] Palmquist, H. , & Hanæus, J. (2005). Hazardous substances in separately collected grey‐ and Blackwater from ordinary Swedish households. Science of the Total Environment, 348(1–3), 151–163. 16162321 10.1016/j.scitotenv.2004.12.052

[wer70096-bib-0064] Paris, S. , & Schlapp, C. (2010). Greywater recycling in Vietnam—Application of the HUBER MBR process. Desalination, 250(3), 1027–1030. 10.1016/j.desal.2009.09.099

[wer70096-bib-0065] Priyanka, K. , Remya, N. , & Behera, M. (2022). Sequential biological and solar photocatalytic treatment system for greywater treatment. Water Science and Technology, 86(3), 584–595. 10.2166/wst.2022.229 35960838

[wer70096-bib-0066] European Commission (2018). Proposal for a regulation of the European Parliament and of the Council on minimum requirements for water reuse, 2018/0169 (COD). Publications Office of the European Union.

[wer70096-bib-0067] QCVN 39:2011/BTNMT (2011). National technical regulation on water quality for irrigated agriculture. Vietnames standard.

[wer70096-bib-0068] Quednow, K. , & Püttmann, W. (2007). Monitoring terbutryn pollution in small rivers of Hesse, Germany. Journal of Environmental Monitoring, 9(12), 1337. 18049772 10.1039/b711854f

[wer70096-bib-0069] Rossmann, J. , Schubert, S. , Gurke, R. , Oertel, R. , & Kirch, W. (2014). Simultaneous determination of most prescribed antibiotics in multiple urban wastewater by SPE‐LC–MS/MS. Journal of Chromatography B, 969, 162–170. 10.1016/j.jchromb.2014.08.008 25171505

[wer70096-bib-0072] Schäfer, A. I. , Nghiem, L. D. , & Oschmann, N. (2006). Bisphenol A retention in the direct ultrafiltration of greywater. Journal of Membrane Science, 283(1–2), 233–243. 10.1016/j.memsci.2006.06.035

[wer70096-bib-0073] Schramm, S. (2016). Hanoi's septic tanks ‐ technology of a city in flow from the late 19th century till today. International planning history society proceedings, 17(4). 10.7480/iphs.2016.4.1300

[wer70096-bib-0074] Sievers, J. C. , & Londong, J. (2018). Characterization of domestic graywater and graywater solids. Water Science and Technology, 77(5), 1196–1203. 10.2166/wst.2017.627 29528307

[wer70096-bib-0075] Stalder, A.‐K. , Patrick, M. , Spitzhofer, N. , Singer, H. , & Burkhardt, M. (2022). Mass balance of diethyltoluamide (DEET) in the environment. Comm. Rep. Swiss Fed. Off. Environ. FOEN.

[wer70096-bib-0076] TCVN (2005). TCVN 5945:2005, Industrial waste water—Discharge standards. Vietnam's industrial wastewater discharge standards.

[wer70096-bib-0077] TCVN (2000). TCVN 6772:2000, Water Quality—Domestic Wastewater Standards. Standards for domestic wastewater quality in Vietnam.

[wer70096-bib-0078] Terasaki, M. , Abe, R. , Makino, M. , & Tatarazako, N. (2015). Chronic toxicity of parabens and their chlorinated by‐products in *Ceriodaphnia dubia* . Environmental Toxicology, 30(6), 664–673. 10.1002/tox.21944 24376163

[wer70096-bib-0079] Thang, P. , Tuan, H. , & Hutton, G. (1995). Economic impacts. Earthquake Spectra, 11(3_suppl), 149–175.

[wer70096-bib-0080] Trang, D. T. , Mølbak, K. , Cam, P. D. , & Dalsgaard, A. (2007). Helminth infections among people using wastewater and human excreta in peri‐urban agriculture and aquaculture in Hanoi, Vietnam. Tropical Medicine & International Health, 12(s2), 82–90. 10.1111/j.1365-3156.2007.01945.x 18005319

[wer70096-bib-0081] TURBOMOLE (2018). TURBOMOLE V7.3 2018, A development of University of Karlsruhe and Forschungszentrum Karlsruhe GmbH, 1989–2007, TURBOMOLE GmbH, since 2007, available from http://www.turbomole.com

[wer70096-bib-0082] Turner, R. (2017). Environmental implications of greywater irrigation within an urban development. Queensland University of Technology.

[wer70096-bib-0083] Ulliman, S. L. , Miklos, D. B. , Hübner, U. , Drewes, J. E. , & Linden, K. G. (2018). Improving UV/H _2_ O _2_ performance following tertiary treatment of municipal wastewater. Environmental Science (Camb), 4(9), 1321–1330.

[wer70096-bib-0084] US EPA . (2012) EPA sustainable futures—P2 framework manual EPA‐748‐B12‐001

[wer70096-bib-0085] WHO . (2006). WHO guidelines for the safe use of wastewater, excreta and greywater: Volume I ‐ policy and regulatory aspects. World Health.

[wer70096-bib-0086] Wick, A. , Marincas, O. , Moldovan, Z. , & Ternes, T. A. (2011). Sorption of biocides, triazine and phenylurea herbicides, and UV‐filters onto secondary sludge. Water Research, 45(12), 3638–3652. 10.1016/j.watres.2011.04.014 21570102

[wer70096-bib-0087] Wright‐Contreras, L. , March, H. , & Schramm, S. (2017). Fragmented landscapes of water supply in suburban Hanoi. Habitat International, 61, 64–74. 10.1016/j.habitatint.2017.02.002

[wer70096-bib-0088] Wu, C. , Spongberg, A. L. , & Witter, J. D. (2009). Adsorption and degradation of triclosan and triclocarban in soils and biosolids‐amended soils. Journal of Agricultural and Food Chemistry, 57(11), 4900–4905. 10.1021/jf900376c 19441835

[wer70096-bib-0089] Wu, C. , Spongberg, A. L. , Witter, J. D. , Fang, M. , & Czajkowski, K. P. (2010). Uptake of pharmaceutical and personal care products by soybean plants from soils applied with biosolids and irrigated with contaminated water. Environmental Science & Technology, 44(16), 6157–6161. 10.1021/es1011115 20704212

[wer70096-bib-0090] Ying, G.‐G. , Yu, X.‐Y. , & Kookana, R. S. (2007). Biological degradation of triclocarban and triclosan in a soil under aerobic and anaerobic conditions and comparison with environmental fate modelling. Environmental Pollution, 150(3), 300–305. 10.1016/j.envpol.2007.02.013 17459543

[wer70096-bib-0091] Zhang, Z.‐F. , Wang, L. , Zhang, X. , Zhang, X. , Li, Y.‐F. , Nikolaev, A. , & Li, W.‐L. (2021). Fate processes of parabens, triclocarban and Triclosan during wastewater treatment: Assessment via field measurements and model simulations. Environmental Science and Pollution Research, 28(36), 50602–50610. 10.1007/s11356-021-14141-9 33963991

