# Supplementary material for: Decentralized graywater treatment by a combination of sequencing batch reactor and advanced oxidation processes for reuse in Vietnam
Source: Water Environ Res. 2025 May 25;97(5):10.1002/wer.70096. doi: 10.1002/wer.70096 (PMC12104610; doi:10.1002/wer.70096)
Supplement: Supplementary file 1 — Table S1. List of chemicals, standards and internal isotopically labeled standards ISTD with their manufacturers or supplier. Figure S1: Image of the SBR in the aeration/agitation phase (left) and settling phase (right). Figure S2: Experimental setup with UV reactor. Table S2. MRMs and retention times for LC–MS/MS analyses of micropollutants. [file WER-97--s001.docx]

**Supplementary Material**

Decentralized Greywater Treatment by a Combination of Sequencing Batch Reactor and Advanced Oxidation Processes for Reuse in Vietnam

AUTHORS:

Stephan Beil^1^, Amélie Chabilan^1^, Linda Schuster^1^, Hilmar Börnick*^,1^, Minh Tan Nguyen^2^, Stefan Stolte^1^

AUTHOR ADDRESS:

^1^Technische Universität Dresden, Chair of Hydrochemistry and Water Technology, 01062 Dresden, Germany

^2^Hanoi University of Science and Technology (HUST) - 1, Institute for R&D of Natural Products (INAPRO) -2, Dai Co Viet, Hanoi, Vietnam

* To whom correspondence should be addressed at the Institute of Water Chemistry, Technische Universität Dresden, Bergstraße 66, 01069 Dresden. E-mail: hilmar.boernick@tu-dresden.de

**Table S1.** List of chemicals, standards and internal isotopically labeled standards ISTD with their manufacturers or supplier

| **Chemical** | **Manufacturers / suppliers** |
| --- | --- |
| ammonium chloride | Grüssing GmbH (Grüssing, Germany) |
| calcium dichloride dihydrate | Grüssing GmbH (Grüssing, Germany) |
| sodium bicarbonate | Grüssing GmbH (Grüssing, Germany) |
| Potassium nitrate | Riedel de Haën (Seelze, Germany), |
| kaolin | Riedel de Haën (Seelze, Germany), |
| cellulose (microcrystalline) | Alfa Aesar GmbH & Co. KG (Karlsruhe, Germany) |
| glycerol | Sigma Aldrich/ Merck KGaA (Darmstadt, Germany) |
| Potassium dihydrogen phosphate | Merck KGaA (Darmstadt, Germany) |
| dipotassium hydrogen phosphate | Merck KGaA (Darmstadt, Germany) |
| sodium sulfate | Merck KGaA (Darmstadt, Germany) |
| sodium dodecyl sulfate (SDS) | Merck KGaA (Darmstadt, Germany) |
| urea | Carl Roth GmbH & Co. KG (Karlsruhe, Germany) |
| disodium hydrogen phosphate | Carl Roth GmbH & Co. KG (Karlsruhe, Germany) |
| humic acid (technical grade) | Acros Organics B.V.B.A (Geel, Belgium) |
| Sodium EDTA | VWR chemicals (Darmstadt, Germany) |
| Magnesium sulfate | VWR chemicals (Darmstadt, Germany) |
| sodium polyphosphate | VWR chemicals (Darmstadt, Germany) |
| sodium chloride | J.T. Baker (Phillipsburg, New Jersey) |
| **Standard / internal isotopically labeled standards** | **Manufacturers / suppliers** |
| chlorpyrifos | HPC Standards GmbH (Borsdorf, Germany) |
| chlorpyrifos-methyl | HPC Standards GmbH (Borsdorf, Germany) |
| N,N-diethyl-meta-toluamide (DEET) | HPC Standards GmbH (Borsdorf, Germany) |
| octinoxate | HPC Standards GmbH (Borsdorf, Germany) |
| methylparaben | HPC Standards GmbH (Borsdorf, Germany) |
| 5,6-dimethyl-1H-benzotriazole | Merck KGaA (Darmstadt, Germany) |
| diclofenac | Merck KGaA (Darmstadt, Germany) |
| terbutryn | Merck KGaA (Darmstadt, Germany) |
| triclosan | Merck KGaA (Darmstadt, Germany) |
| triclocarban | Merck KGaA (Darmstadt, Germany) |
| bisphenol A | Merck KGaA (Darmstadt, Germany) |
| bisphenol A-d16 | Merck KGaA (Darmstadt, Germany) |
| benzylparaben | Merck KGaA (Darmstadt, Germany) |
| phenylparaben | Merck KGaA (Darmstadt, Germany) |
| isoamyl 4-methoxycinnamate | Merck KGaA (Darmstadt, Germany) |
| 1H‑benzotriazole | LGC Standards GmbH (Wesel, Germany) |
| triclosan-d3 | LGC Standards GmbH (Wesel, Germany) |
| triclocarban-d4 | CDN isotopes (Pointe-Claire, Canada) |
| diclofenac-d4 | NEOCHEMA GmbH (Bodenheim, Germany) |
| terbutryn-d5 | NEOCHEMA GmbH (Bodenheim, Germany) |
| DEET-d7 | NEOCHEMA GmbH (Bodenheim, Germany) |

**Sequencing batch reactor:**


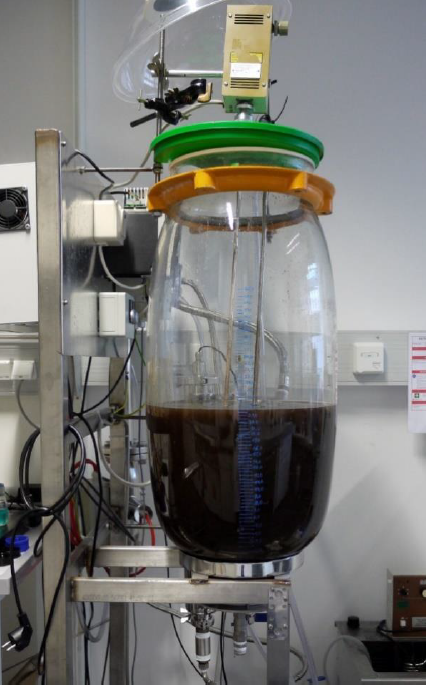

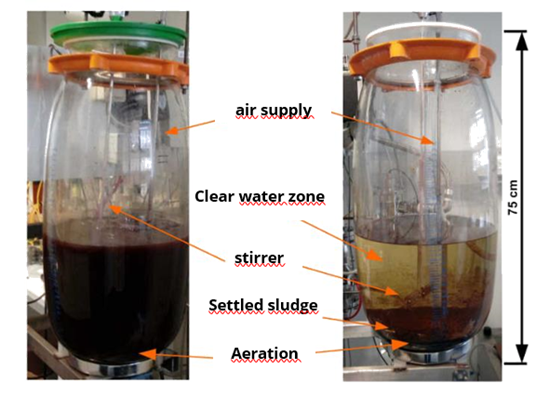


**Figure S1:** Image of the SBR in the aeration/agitation phase (left) and settling phase (right)

***Additional Information on the used SBR:***

An aquarium aerator was installed in the reactor to supply oxygen through compressed air. The dead volume (1 L) at the reactor floor was also aerated by means of attached aquarium stones and adjusted to approx. 0.25 bar fine bubbles via the controller. The outlet valve below the reactor is used to remove excess sludge. Furthermore, a peristaltic pump was installed to take samples of the clarified water. A measuring scale was applied to the reactor wall to determine the volume of the reactor content. To reduce evaporation losses, the reactor was covered with a specifically adapted and removable plastic lid.

**Laboratory-scale reactor UXPM-LAB-400:**

**Figure S2:** Experimental setup with UV reactor

***Technical details***

Manufacturer: UV-EL GmbH & Co.KG

Components: UV reactor with protective tube, control panel, UV lamp

*UV-reactor*

Material: stainless steel 1.4301, electropolished on the outside, pickled on the inside

Dimensions: Reactor diameter: 114 mm, Reactor length: 500 mm (total)

Flange distance: 260 mm

Permissible operating pressure: 10 bar

Pressure loss: approx. 0.1 bar

Material of protective tube: quartz, UVC permeability 90 %/ 1 mm (254 nm)

*UV lamp*

Medium pressure lamp: max. 400 W

Lamp service life: 1,500 – 3,000 h (depending on effective power and number of switch operations)

Spectrum: UVC range (200 – 280 nm) W 50, UVB range (280 – 315 nm) W 20, UVA range (315 – 400 nm) W 20

**Table S2.** MRMs and retention times for LC-MS/MS analyses of micropollutants

| Compound MRM | ESI mode | Q1 m/z | Q3 m/z | retention time [min] |
| --- | --- | --- | --- | --- |
| **Analytes** |  |  |  |  |
| methylparaben 1 | negative | 150.970 | 91.700 | 2.61 |
| methylparaben 2 | negative | 150.970 | 136.000 | 2.61 |
| benzylparaben 1 | negative | 227.008 | 91.800 | 3.29 |
| benzylparaben 2 | negative | 227.008 | 135.900 | 3.29 |
| triclosan 1 | negative | 286.683 | 34.900 | 4.11 |
| triclosan 2 | negative | 288.705 | 34.900 | 4.11 |
| triclocarban 1 | negative | 312.912 | 159.800 | 4.10 |
| triclocarban 2 | negative | 312.912 | 126.000 | 4.10 |
| octinoxate 1 | positive | 291.091 | 160.900 | 5.05 |
| octinoxate 2 | positive | 291.091 | 179.000 | 5.05 |
| 1H-benzotriazole 1 | positive | 119.997 | 65.100 | 2.29 |
| 1H-benzotriazole 2 | positive | 119.997 | 92.000 | 2.29 |
| bisphenol A 1 | negative | 226.761 | 212.100 | 3.01 |
| bisphenol A 2 | negative | 226.761 | 133.000 | 3.01 |
| diclofenac 1 | negative | 293.906 | 250.000 | 3.69 |
| diclofenac 2 | negative | 295.897 | 252.000 | 3.69 |
| DEET 1 | positive | 192.090 | 119.000 | 3.07 |
| DEET 2 | positive | 192.090 | 90.900 | 3.07 |
| chlorpyrifos 1 | positive | 349.879 | 197.900 | 4.59 |
| chlorpyrifos 2 | positive | 349.879 | 321.700 | 4.59 |
| terbutryn 1 | positive | 242.069 | 186.000 | 3.52 |
| terbutryn 2 | positive | 242.069 | 90.900 | 3.52 |
| **ISTDs** |  |  |  |  |
| phenylparaben | negative | 212.956 | 92.900 | 3.20 |
| triclosan-d3 | negative | 289.683 | 34.900 | 4.11 |
| triclocarban-d4 | negative | 316.916 | 159.800 | 4.10 |
| isoamly 4-methoxycinnamate | positive | 249.072 | 179.000 | 4.35 |
| 5,6-dimethyl benzotriazole | positive | 148.053 | 77.000 | 2.64 |
| bisphenol A-d16 | negative | 241.084 | 141.900 | 3.01 |
| diclofenac-d4 | negative | 299.900 | 256.000 | 3.69 |
| DEET-d7 | positive | 199.100 | 126.100 | 3.07 |
| chlorpyrifos-methyl | positive | 321.913 | 125.000 | 4.17 |
| terbutryn-d5 | positive | 247.094 | 191.000 | 3.52 |
|  |  |  |  |  |
